# From pathogenesis to antigens: the key to shaping the future of TB vaccines

**DOI:** 10.3389/fimmu.2024.1440935

**Published:** 2024-07-23

**Authors:** Haoyan Yang, Xinkui Lei, Siyu Chai, Guimin Su, Lin Du

**Affiliations:** ^1^ Research and Development Centre, Beijing Zhifei Lvzhu Biopharmaceutical Co., Ltd., Beijing, China; ^2^ Beijing Bacterial Vaccine Engineering Research Centre, Beijing, China

**Keywords:** tuberculosis, *M. tuberculosis*, pathogenesis, immune evasion, vaccine

## Abstract

Tuberculosis (TB) remains one of the gravest global health challenges. *Mycobacterium tuberculosis* (*M. tuberculosis*), the causative agent, employs sophisticated immune evasion and pathogenesis strategies. Its capability to thrive within immune cells and incite robust inflammatory responses prolongs infection and dissemination. Mycobacterial advanced adaptations facilitate navigation through the human immune system and present a variable antigenic profile throughout different infection stages. Investigating these strategies unfolds targeted approaches to effective vaccine development against TB. This review delves into the most advanced and exhaustive insights into the immune evasion tactics and pathogenic processes of *M. tuberculosis* across various infection stages. The knowledge distilled from this analysis holds the promise of guiding the creation of innovative TB vaccines and translating theoretical groundwork into practical immunological defenses.

## Introduction

In the landscape of global health threats, TB, driven by the virulent *M. tuberculosis*, firmly stood as a leading cause of infectious mortality, second only to the COVID-19 pandemic in 2022 (1). Despite concerted public health efforts, TB continues to afflict over 10 million individuals annually (1), underscoring the disease’s persistence and reach. The concerning prevalence of latent TB infection, impacting approximately 25% of the global population, exemplifies the insidious nature of *M. tuberculosis* and the daunting challenge it poses (2). Currently, the Bacillus Calmette-Guérin (BCG) vaccine is the sole prophylactic measure authorized for TB, yet it provides limited protection in the adult population. The discrepancy between the vast scale of infection and the constrained efficacy of existing vaccines and diagnostics highlights a compelling need for rigorous scientific inquiry into novel detection and prevention modalities. There is a paramount need for the development and deployment of innovative, effective strategies that enhance our capacity to accurately identify *M. tuberculosis* infections and fortify our preventive measures. Embracing novel diagnostic technologies, alongside a fervent pursuit of vaccine research that aims to transcend the constraints of current options, is indispensable.

*M. tuberculosis*, the etiological agent of TB, manifests a complex array of biological traits that critically impede its prevention and management. Notably, *M. tuberculosis* is characterized by a slow replication rate, typically requiring 24 hours to divide in artificial culture media or within diseased animal models, which is an attribute that underpins TB’s chronicity and necessitates comprehensive, long-term treatment regimens. This inherent slowness of growth presents a substantial hurdle for researchers and clinicians alike. Furthermore, *M. tuberculosis* has evolved the capacity for dormancy. Within the infected tissues, it can enter a state of metabolic stasis induced by the host immune response. While this dormancy curtails the progression of active infection, it is insufficient for pathogen eradication. Significantly, this latent reservoir of bacteria can become reactivated and pathogenic once host immunity diminishes due to either the natural aging process or immunosuppression, often culminating in a resurgence of the disease. This dynamic has positioned TB prevention and treatment at the forefront of modern medical challenges. Central to the endeavor of combating TB is the elucidation of the mechanisms underpinning immune recognition and response to *M. tuberculosis*. A sophisticated comprehension of infection dynamics, immune cell functionality, enhancement of host immune defenses, and the subterfuges *M. tuberculosis* employs to escape immune surveillance is imperative. This review delineates current knowledge about the immunological landscape of TB, with an emphasis on delineating the complex life cycle of *M. tuberculosis*, its pathogenic processes, and sophisticated immune evasion strategies. It also concentrates on the imperative of TB vaccine development, spotlighting the identification of immunodominant antigens as a cornerstone strategy for pioneering therapeutics.

## Earliest events of *M. tuberculosis* infection

TB transmission is primarily facilitated by aerosols containing *M. tuberculosis*, expelled into the air by individuals with active TB during activities such as coughing, sneezing, or speaking (3). The potential for *M. tuberculosis* to transmit from human to animal has also been documented, with an early account by Hermann Tappeiner in Germany, who in 1878 described the transmission of TB to a dog via inhalation of aerosolized sputum from a TB patient (4). This observation by Tappeiner is possibly the earliest recorded evidence of the transmission route of *M. tuberculosis* (4). *M. tuberculosis* is notably highly infectious, with the infectious dose likely being as low as a single organism (5).

Upon inhalation, *M. tuberculosis* encounters the alveolar lining fluid, a critical component of the lung’s innate defenses. This fluid is rich in soluble factors, including antimicrobial hydrolytic enzymes (6), which can disrupt the *M. tuberculosis* cell envelope. The presence of both *M. tuberculosis* and its envelope fragments can enhance the bactericidal activity of macrophages (7). Additionally, these hydrolases can degrade several key components of the cell envelope, such as the carbohydrates arabinose, mannose (Man), and glucose (Glc), among others (6). These carbohydrates play a crucial role in the recognition of *M. tuberculosis* by macrophages, the regulation of mycobacterial intracellular survival, and the overall pathogenesis of TB (8, 9).

Treatment of *M. tuberculosis* with alveolar lining fluid has been shown to significantly reduce interactions between the bacterium and macrophages, as well as restrict the intracellular proliferation of the bacteria within macrophages (6, 10). However, this reduced interaction and recognition by macrophages might paradoxically increase the pathogenicity of *M. tuberculosis*. Intriguingly, the survival capability of *M. tuberculosis* does not appear to be compromised by the absence of its cell envelope, and *M. tuberculosis* treated with alveolar lining fluid demonstrates a marginally faster growth rate compared to untreated cells (6).

## *M. tuberculosis* invasion of alveolar epithelial cells

Infection occurs in alveolar epithelial cells within 48 hours. In the early stages of infection, *M. tuberculosis* demonstrates a specific affinity for invasion and replication within alveolar epithelial type II cells (ATII) typically within 48 hours post-inhalation (11–14). These cells, although non-phagocytic by nature, may inadvertently provide a conducive environment for bacterial proliferation, essentially serving as a sanctuary that facilitates *M. tuberculosis* growth (15, 16). Moreover, this localization potentially allows *M. tuberculosis* to gain direct entry into the host’s lymphatic and circulatory systems, bypassing the need for transport by carrier macrophages (12, 17).

The involvement of a heparin-binding hemagglutinin (HBHA) expressed by *M. tuberculosis* has been proposed to mediate the pathogen’s adherence and subsequent internalization into these nonprofessional phagocytes (16). Investigative work by Pethe et al. demonstrated the functional significance of HBHA; deletion of the *hbhA* locus in *M. tuberculosis* did not impair bacillary growth per se but resulted in a significant reduction in the invasion of alveolar epithelial cells, while phagocytic interactions remained unaltered (18). Additionally, though equivalent colonization rates were observed in the lungs of mice infected with wild-type and *ΔhbhA M. tuberculosis* strains, the latter exhibited a profound deficit in dissemination to distal organs (18).

**Figure 1:**
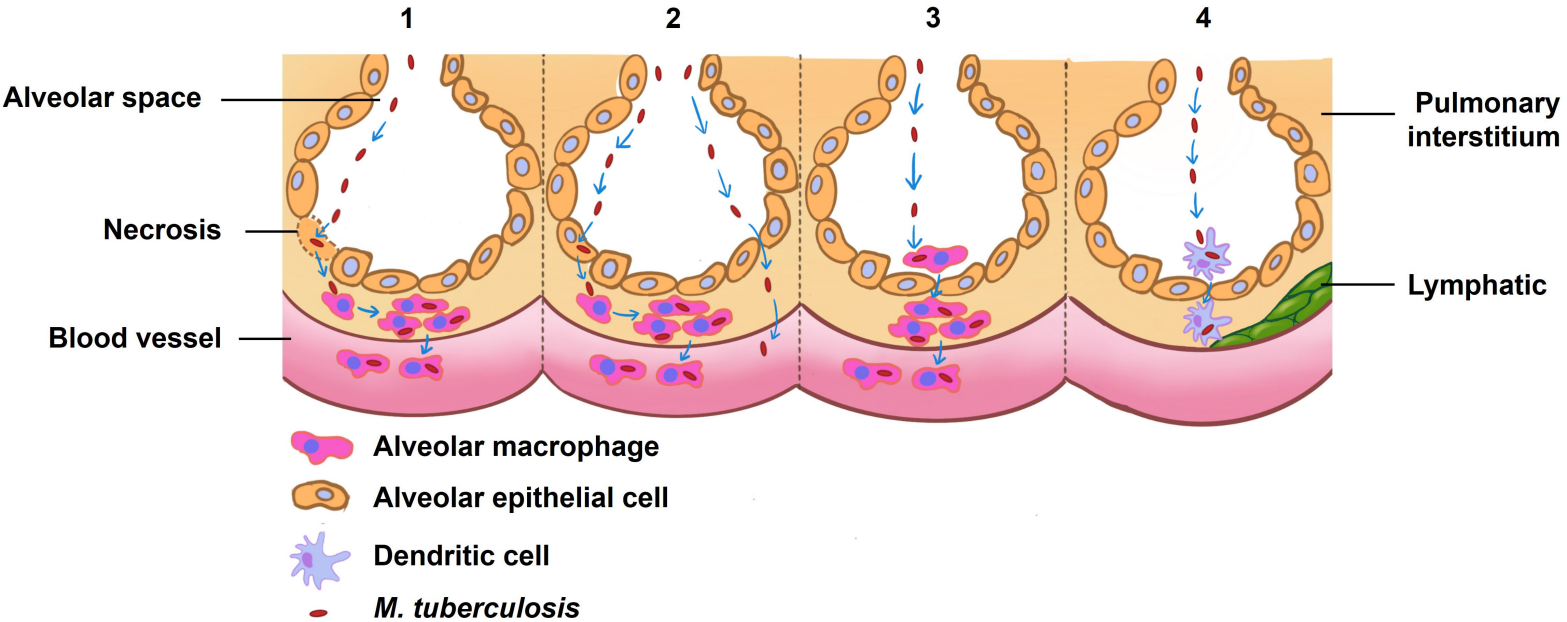
Theories of how inhaled *M. tuberculosis* might cause an infection in the lung and spread to other areas. The first proposed model delineates a direct binding of *M. tuberculosis* to alveolar epithelial cells, culminating in cellular lysis, which triggers an inflammatory cascade and consequent tissue damage, thereby paving a pathway for hematogenous spread. In an alternate scenario, *M. tuberculosis* gains entry into alveolar epithelial cells and, through transcytosis, traverses these cells to either directly invade macrophages or access the vasculature. A third hypothesis posits that alveolar macrophages, upon phagocytosing *M. tuberculosis*, migrate to the pulmonary interstitium and vascular systems, serving as vectors for the pathogen’s mobility. The final conjecture suggests that dendritic cells resident in the alveoli may capture *M. tuberculosis* and subsequently transport it to proximal lymph nodes. Collectively, these models underpin our understanding of the initial pulmonary infection dynamics and the potential systemic spread of *M. tuberculosis*, crucial for informing the development of targeted interventions.

Post-entry into the alveolar epithelial cells, *M. tuberculosis* propagation ensues (12, 13, 17, 19). There are four posited mechanisms by which inhaled *M. tuberculosis* may incite pulmonary infection and orchestrate systemic spread (refer to **Figure 1**) (16). The first model involves the mycobacteria directly binding and entering alveolar epithelial cells, which then lyse to cause tissue damage and an inflammatory reaction. This process aids in the spread of the bacteria into the bloodstream. The second scenario entails the bacteria attaching and entering into alveolar epithelial cells, transcytosis through the epithelial cells, and being delivered to macrophages or accessing the blood vessels themselves. The third theory proposes that after *M. tuberculosis* infects alveolar macrophages, the infected macrophages move into the lung interstitium, where they trigger an inflammatory response that draws in more immune cells (16). Additionally, dendritic cells (DCs) have been speculated to play an instrumental role in mycobacterial dispersal. Research by Reljic et al. underscores that DCs migrate into the pulmonary system and phagocytose *M. tuberculosis* in less than 48 hours (20), thereafter relocating to lung-draining lymph nodes, priming T cells for antigen recognition (21, 22).

For successful infection establishment, mycobacteria must circumvent anti-bacterial monocyte responses. A genetic variant screen of *Mycobacterium marinum* identified the cell-surface lipid phthiocerol dimycocerosate (PDIM) as vital for eluding such responses (23). PDIM can infiltrate the alveolar epithelial barrier and recruit permissive monocytes—their movement is orchestrated by the chemokine CCL2, distinct from the Toll-like receptor/myeloid differentiation factor 88 (TLR/Myd88)-dependent monocytes typified by anti-bacterial attributes (24). The unique methyl-branched fatty acids present in PDIM are also thought to increase lipid fluidity within membranes, thereby promoting bacterial dissemination (24).

## Alveolar macrophages create an early niche for *M. tuberculosis*

Fourteen days following aerosol exposure, alveolar macrophages (AMs) emerge as the primary reservoirs of *M. tuberculosis* within the pulmonary tissue of murine models (25). These macrophages are not mere passive hosts; rather, they are pivotal in shuttling *M. tuberculosis* from the alveoli to the lung interstitium and subsequently to monocytes, which are amenable to bacillary replication. Research indicates that the eradication of AMs correlates with a marked reduction in mycobacterial burden (26, 27).

The transition of *M. tuberculosis*-infected AMs from the alveolar space into the lung interstitium is contingent upon two critical elements: MyD88/IL-1 signaling and the ESX-1 secretory system (25). Interleukin-1 (IL-1), a versatile cytokine, is implicated in the modulation of vascular permeability, angiogenesis, and the inflammatory response to infectious agents (28, 29). In the context of TB infection, IL-1 has been identified as having an intricate protective function. MyD88 serves as a universal adaptor molecule that connects the IL-1 receptor and Toll-like receptors (TLRs) to downstream signaling pathways. Activation of MyD88/IL-1 signaling in alveolar epithelial cells contributes to enhanced alveolar permeability, thereby allowing the influx of immune cells such as neutrophils and facilitating the transit of infected AMs across the alveolar epithelium into the lung interstitium (25).

**Figure 2:**
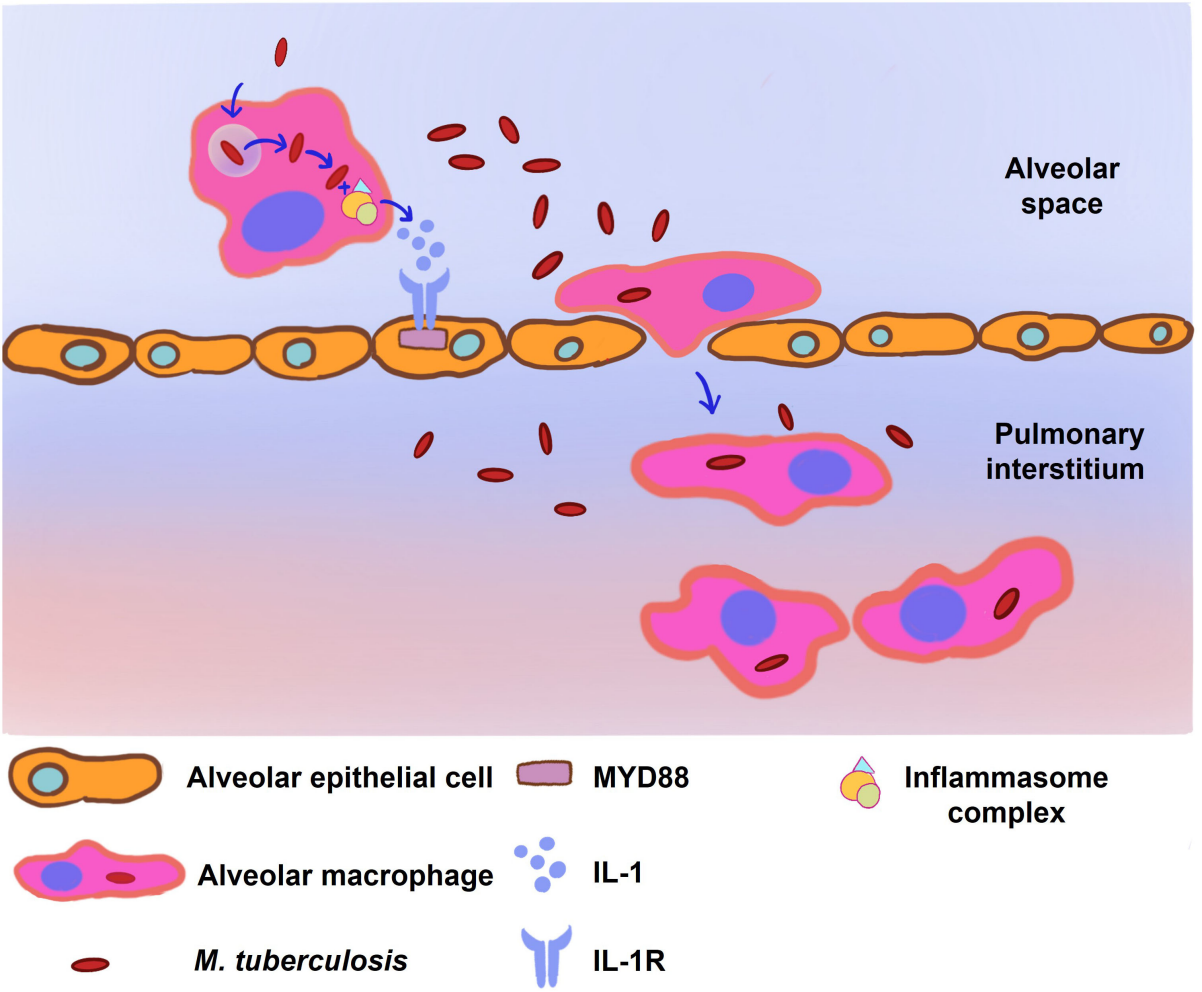
The infected alveolar macrophages (AM) stimulate the MyD88/IL-1 signaling pathway to facilitate their translocation to the pulmonary interstitium. Following *M. tuberculosis* infection, the ESX-1 secretion system of *M. tuberculosis* orchestrates the disruption of phagosomal membranes within the AM, a critical event facilitating mycobacterial entry into the macrophage cytoplasm. This invasion triggers the assembly of the NLRP3, ASC, and caspase-1 inflammasome complex, resulting in a surge of cytokine production, notably interleukin-1 (IL-1). IL-1, in turn, activates the MyD88/IL-1 signaling cascade in neighboring alveolar epithelial cells, leading to enhanced alveolar permeability. The culmination of these molecular events facilitates the migration of the infected AMs from the alveolar space into the pulmonary interstitium, underscoring a crucial step in the progression of *M. tuberculosis* infection within the lung parenchyma.

The ESX-1 system, encoded by the region of difference 1 (RD1) locus of *M. tuberculosis*, has been characterized as a virulence determinant essential for phagosomal escape, enabling the bacteria to gain access to the macrophage cytosol (30). Early secreted Ag of 6 kDa (ESAT-6), one of the components secreted by the ESX-1 system, augments the migration of *M. tuberculosis* from the phagosome to the cytoplasm (30–33). The cytosolic presence of ESAT-6 is implicated in the activation of the NLRP3 inflammasome complex, including ASC and caspase-1, a process instrumental for the liberation of IL-1 (**Figure 2**) (32). Concurrently, there is evidence suggesting that the activation of NLRP3 could facilitate further bacterial egress from the phagosome by instigating necrotic cell death, a phenomenon that appears to be TLR2-MyD88 dependent (33–35).

## Eventually persistent infection develops in the pulmonary interstitium

### *M. tuberculosis* invasion of macrophages

As mycobacterial infection progresses to the pulmonary interstitium, a pivotal phase in the establishment of a persistent infection unfolds through its invasion of macrophages. The infiltration of AMs into the lung interstitium results in the subsequent recruitment and infection of additional macrophage populations. To counteract *M. tuberculosis* invasion, macrophages deploy a repertoire of defense strategies, including phagosome-lysosome fusion to degrade pathogens, recruitment of hydrolytic lysosomal enzymes, production of reactive oxygen species (ROS) and reactive nitrogen species (RNS), antigen presentation, upregulation and mobilization of major histocompatibility complex class II (MHC class II) molecules, autophagy, and induction of apoptosis (36). Studies have shown that selective ablation of distinct macrophage subtypes escalates the pulmonary *M. tuberculosis* load in murine models, underscoring the pivotal role of monocyte-derived macrophages in containing *M. tuberculosis* (26).

While AMs are often the initial phagocytes to encounter *M. tuberculosis*, their ability to impede intracellular *M. tuberculosis* proliferation seems compromised compared to interstitial macrophages (IMs), which exhibit a more robust bacteriostatic effect (26, 27). *M. tuberculosis* exhibits a higher replication rate and a reduced stress response within AMs. Bacterial burden is decreased when AM is depleted, whereas it is elevated when IM is depleted, highlighting macrophages’ differential roles and reiterating the importance of IMs in infection control (26). Notably, IMs possess more enduring stability and a shorter lifespan than AMs (37).

Divergent metabolic pathways are implicated in the variations observed in the bactericidal capabilities of different macrophage populations. Transcriptomic analyses reveal that AMs exhibit augmented fatty acid uptake and β-oxidation, whereas IMs are predominantly glycolytic (26). Given that *M. tuberculosis* intracellularly harvests cholesterol and fatty acids from the host cell, it’s plausible that the metabolic pathways active in AMs confer a nutritive edge to *M. tuberculosis* (38–41). Additionally, the interplay between macrophages and CD4^+^ T cells is critical for the containment of *M. tuberculosis*. It is suggested that macrophages exerting a restrictive impact on *M. tuberculosis* are those that have engaged effectively with CD4^+^ T cells, emphasizing the reliance of macrophage-mediated control of *M. tuberculosis* on this cellular interaction.

### Strategies of *M. tuberculosis* for immune evasion

**Figure 3:**
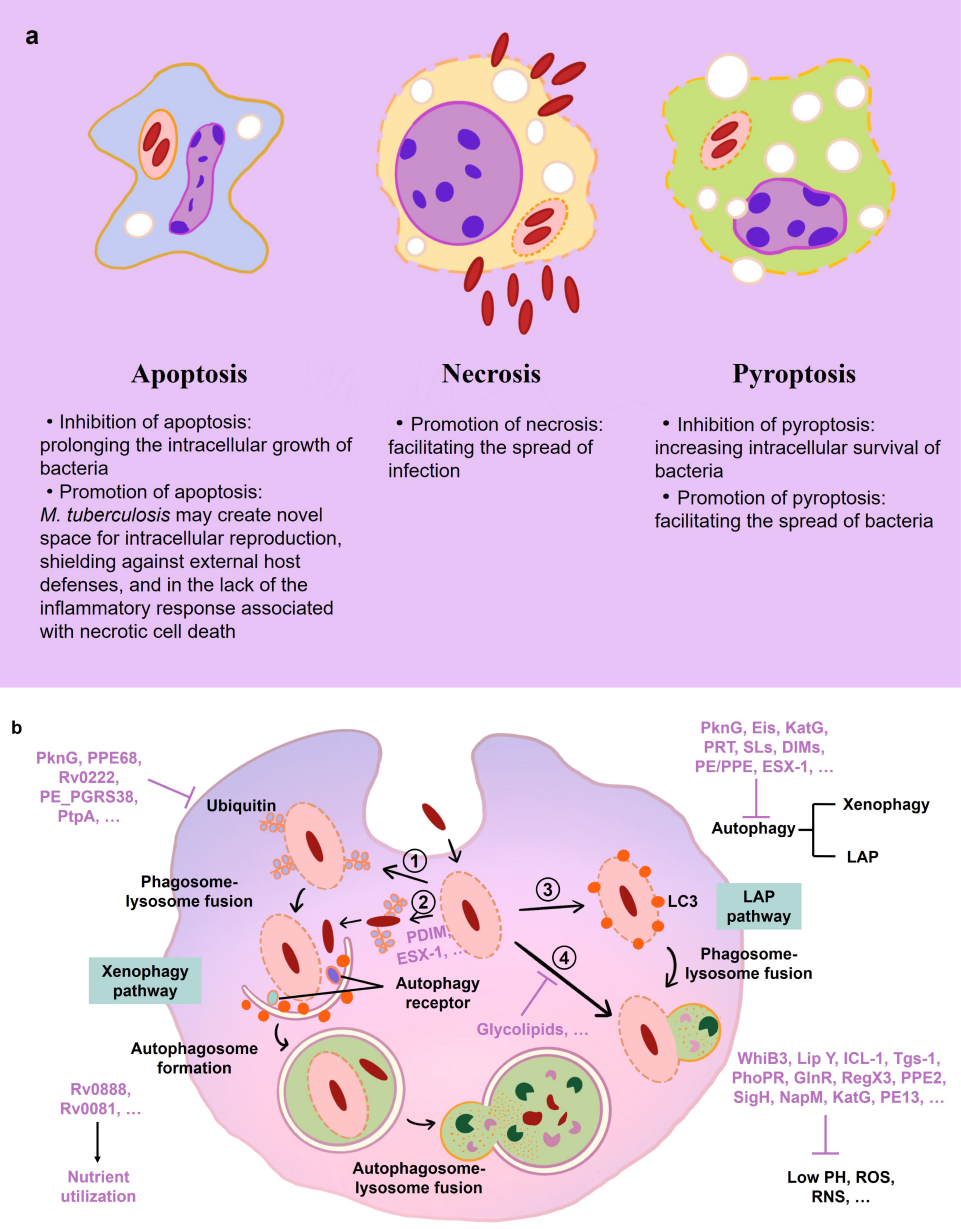
Immune events in macrophage during *M. tuberculosis* infection. **(A)** Mechanisms of host cell death during *M. tuberculosis* infection. **(B)** Mechanisms of mycobacterial immune evasion in macrophage. *M. tuberculosis* is absorbed in phagosomes. WhiB3, Lip Y, ICL-1, and other *M. tuberculosis* factors (purple) aid in the bacilli’s resistance to low pH, ROS, and RNS. PDIM and ESX-1 encourage phagosomal damage. (1, 2) Ubiquitin (Ub) ligases bind the phagosome and *M. tuberculosis* to attract autophagy receptors, which is blocked by PknG, PPE68 and other factors. The phagophore membrane interaction of LC3 and its subsequent fusion with the lysosome directs *M. tuberculosis* toward xenophagy. (3) The LC3-associated phagocytosis (LAP) pathways are involved in the macrophage autophagy-mediated elimination of *M. tuberculosis*. (4) Mycobacteria-containing phagosomes fuse to lysosomes, which is inhibited by the glycolipids of *M. tuberculosis*. Rv0888 and Rv0081 help *M. tuberculosis* with the utilization of nutrients in macrophage.

*M. tuberculosis* employs a suite of sophisticated strategies to evade the host immune system, enabling the pathogen to sustain a chronic infection despite the robust inflammatory response mounted by IMs (refer to **Figure 3** and **Table 1** for an overview of evasion mechanisms). Upon phagocytosis, *M. tuberculosis* faces an oxidative onslaught, as macrophages initiate a respiratory burst aiming to obliterate the bacilli. Remarkably, *M. tuberculosis* demonstrates resilience against DNA damage induced by oxidative stress, succumbing only under conditions of extreme oxidative stress (91). An alternative sigma factor, SigH, plays a pivotal role in enabling *M. tuberculosis* to re-establish oxidative equilibrium following exposure to oxidative stress (79). During the macrophage respiratory burst, reactive oxygen species (ROS) and nitrogen intermediates are generated. The robust cell wall of *M. tuberculosis* efficiently mitigates ROS damage (97), and the enzymes katG and trxB2—whose expression is significantly upregulated in response to H_2_O_2_ and NO—contribute to mycobacterial resistance against oxidative reactions (91, 98).

**Table 1:** List of antigens by which *M. tuberculosis* overcomes killing by macrophage.

Overview	Effector	Mechanism	References
Impairing the integrity of membrane compartments	PDIM	PDIM together with ESX-1 cause phagosomal membrane damage and the rupture of macrophage	(42)
Inhibition of host-mediated ubiquitination	Protein kinase G (PknG)	PknG functions as an atypical ubiquitinating enzyme to inhibit ubiquitination in macrophage	(43)
	PPE68/Rv3873	PPE68 inhibits NF-κB and AP-1 signaling activation as well as the generation of TNF-α, IL-6, and NO via interacting with macrophage ubiquitin ligase (E3)	(44)
	Rv0222	Through interaction with the host E3 ubiquitin ligase ANAPC2, Rv0222 prevents ubiquitination and activation of TRAF6 by facilitating the binding of the protein tyrosine phosphatase SHP1 to the adaptor protein TRAF6	(45)
	PE_PGRS38	PE_PGRS38 binds to herpesvirus-associated ubiquitin-specific protease (HAUSP, USP7) and modifies the level of ubiquitination of diverse substrate proteins	(46)
Inhibition of autophagy	PknG	PknG enhances the initiation of autophagy but suppresses autophagosome maturation, which results in a general blockage of autophagy flux and increased intracellular survival of pathogens	(47)
	Eis	Mycobacteral Eis performs crucial functions in controlling macrophage autophagy, inflammatory reactions and cell death through a mechanism reliant on reactive oxygen species (ROS) and up-regulation of IL-10	(48, 49)
	Phosphoribosyltransferase (PRT)	To increase intracellular bacterial viability, PRT promotes histone hypermethylation in genes relevant to autophagy, which suppresses autophagy	(50)
	Sulfoglycolipids (SLs) and phthiocerol dimycocerosates (DIMs)	Various strategies are used by SLs and DIMs in human macrophages to regulate autophagy-related processes	(51)
	PE/PPE protein family	PE/PPE proteins suppress autophagy and improve intracellular bacterial survival by enhancing mTOR activity and lowering production of TNF-α and IL-1β	(52)
	PE/PPE protein family	PE_PGRS20 and PE_PGRS47 interacted with the Ras-related protein Rab1A in order to prevent the beginning of autophagy	(53)
	PE/PPE protein family	By reducing the activity of extracellular signal-regulated kinase 1/2 (ERK1/2), PPE51 inhibits autophagy and impedes bacterial phagocytosis	(54)
Inhibition of apoptosis	PknE	PknE, which increases macrophage viability through blocking apoptosis, is crucial for *M. tuberculosis* survival	(55)
	SigH	SigH may increase the pathogen’s persistence by lowering the apoptosis of infected monocyte	(56)
	Mannose-capped lipoarabinomannan (ManLAM)	ManLAM-dependent suppression of macrophage apoptosis is achieved by upregulating antiapoptotic B-cell CLL/lymphoma 2 (Bcl2) family member A1	(57)
	Cpn60.2	Cpn60.2 prevents macrophage apoptosis by interacting with host mortalin	(58)
	PE_PGRS18	PE_PGRS18 primarily increases pathogen survival in macrophage by reducing macrophage apoptosis	(59)
	PE_PGRS62	By interfering with ER stress-mediated apoptosis, PE_PGRS62 increases the survival of mycobacteria in macrophage	(60)
	PE31 (Rv3477)	PE31 increases GTPase guanylate binding protein-1, which attenuates macrophage apoptosis and facilitates mycobacteria staying within cells	(61)
	PPE10 (Rv0442c)	PPE10 uses the linear ubiquitin chain assembly complex HOIP-NF-κB signaling axis to suppress macrophage apoptosis	(62)
	Rv3033	In macrophage, mycobacteria-secreted virulence factor Rv3033 effectively thwarts mycobacteria-induced early and late apoptosis	(63)
Promotion of apoptosis	ESAT-6	ESAT-6-induced apoptosis facilitates the transmission of bacteria from cell to cell	(64)
	PPE32	PPE32 triggers macrophage apoptosis and reduces macrophage cell viability	(65)
Promotion of necrosis	Bcl-2	Bcl-2 causes macrophage necrosis and prevents apoptosis	(66)
	Bcl-x_L_	Bcl-x_L_ causes necrosis by triggering the action of RIPK3 and blocking the activation of caspase 8	(67)
Inhibition of pyrotosis	PknF	By inhibiting the NLRP3 inflammasome and pyroptosis, PknF of *M. tuberculosis* plays a significant role in innate immune evasion	(68)
Promotion of pyrotosis	Rv3361c-Rv3365c	Rv3361c-Rv3365c inhibit macrophage pyroptosis through a process involving cytoplasmic surveillance proteins	(69)
	ESX-1	ESX-1 promotes macrophage pyroptosis, which is mediated by caspase-1/NLRP3/gasdermin D	(70)
Cytokines, necrosis, apoptosis, autophagy	PE_PGRS41	PE_PGRS41 enhances macrophage necrosis while suppressing apoptosis and autophagy and lowering the synthesis of TNF-α, IL-1β, and IL-6	(71)
Ubiquitination, apoptosis	PtpA	PtpA inhibits ubiquitination dependent on the pathways of Jnk and p38 as well as NF-κB	(72)
		PtpA inhibits the activation of the JNK/p38 MAPK pathway and cell apoptosis that TRIM27 promotes	(73)
Survival under stress	WhiB3	WhiB3 combines with critical host gases and metabolic signals to keep the redox balance	(74)
	Lip Y, ICL-1, Tgs-1	Lip Y, ICL-1, and Tgs-1 are proteins involved in lipid metabolism for *M. tuberculosis* to resist the oxidative stress caused by macrophages	(74)
	PhoPR	Activation of WhiB3 by PhoPR aids mycobacterial resistance to macrophage-caused low pH	(75)
	GlnR	By directly triggering the expression of whiB3, the nitrogen regulator GlnR regulates SL-1 lipid anabolism and cell adaptability to redox stress	(76)
	RegX3	The cytosolic redox sensor WhiB3 is activated by RegX3 to resist low pH	(77)
	PPE2	PPE2 suppresses NADPH oxidase-mediated ROS production in macrophage to promote *M. tuberculosis* survival	(78)
	SigH	SigH is crucial for *M. tuberculosis* to restore oxidative equilibrium after oxidative stress	(79)
	NapM	NapM prevents mycobacterial DNA synthesis and shields the bacteria from dying under stress	(80)
Lipid metabolism	Rv0888	Rv0888 has strong sphingomyelinase activity that cleaves sphingomyelin, a key lipid in eukaryotic cells, into phosphocholine and ceramide, which are used by *M. tuberculosis* as a source of several vital nutrients	(81)
	Rv0081	Rv0081 facilitates mycobacterial utilization of cholesterol, subversion of lysosomal trafficking and formation of granulomas	(82)
Promotion of host-pathogen interaction	Peptidyl prolyl isomerase A (PPiA)	PPiA interacts with the host integrin receptor and causes granuloma-like lesions in mice, which accelerates the progression of the disease	(83)
Inhibition of inflammasome activation	Zmp1	Zmp1 blocks the processing of IL-1β and the activation of inflammasomes, which inhibits macrophage elimination of pathogens	(84)
Inhibition of inflammatory cytokines	Mycolic acid	Mycolic acid binds to TREM2 on macrophages, inhibiting their ability to fight bacteria	(85)
Multiple mechanisms	ESX-1	ESX-1 substrates are associated with mycobacteria’s ability to damage membranes	(86, 87)
		Members of the ESX-1 system inhibit autophagy in order to facilitate mycobacteria escape into the cytoplasm	(88, 89)
		ESX-1 leads to host cell apoptosis	(42)
	KatG	KatG decreases lysosomal delivery to the phagosome and is essential for the *M. tuberculosis* Beijing strain to avoid starvation-induced autophagic constraint	(90)
		*M. tuberculosis* detoxifies H_2_O_2_ with the aid of KatG	(91)
	DosS	DosS is involved in the inhibition of TNF-α and autophagy pathways and is necessary for mycobacterial replication	(92)
	Rv1515c	Rv1515c leads to increased bacterial tolerance to several stresses and increased cellular survival in macrophage	(93)
		Rv1515c reduces antigen presentation by lowering the expression of MHC-I/MHC-II and co-stimulatory signals, decreasing phagolysosomal maturation and modulating pro-inflammatory cytokine production	(94)
	Mycobacterial glycolipids	The macrophage response is hindered by the glycolipids in the mycobacterial envelope, which bind to TLR2 on the macrophage to prevent NF-κB activation and production of cytokines and costimulatory molecules	(95)
	PE13 (Rv1195)	PE13 facilitates macrophage apoptosis in the late stage of infection	(96)
		PE13 increases bacteria survival in stressful environments such low pH, SDS, and H_2_O_2_	(96)

The disruption of phagolysosomal fusion, a seminal strategy utilized by *M. tuberculosis*, was documented as early as 1971 (99). *M. tuberculosis* disrupts cellular compartment integrity, including macrophage membranes, phagosomes, and phagolysosomes, through mechanisms dependent on ESX-1 and PDIM (42, 86, 87, 100). ESX-1, part of the *M. tuberculosis*-specific type VII secretion system (encompassing ESX-1–5), plays a crucial role in this process (87, 101). The type VII secretion system’s impairment is associated with the attenuation observed in the *Mycobacterium bovis* BCG strain (30, 101, 102). Membrane perforation is facilitated by the primary effector of the ESX-1 system, ESAT-6 (ESX-A), and its substrate, CFP-10 (ESX-B), while ESX system components ESX-3, ESX-H, and ESX-G prevent phagolysosome maturation (103–107).

Furthermore, *M. tuberculosis* manipulates autophagy and cell death pathways to evade innate immune detection (108–114). Autophagy, the process by which cytoplasmic components are degraded or recycled, is inhibited by members of the ESX-1 system, permitting mycobacterial escape into the cytoplasm (88, 115). ESX-A (ESAT-6) is implicated in inducing necrosis in infected host cells, a mechanism that facilitates bacterial dissemination and hinders macrophage containment of the infection (89, 103, 116). Consequently, *M. tuberculosis* has evolved multiple evasion tactics, including oxidative stress resistance, inhibition of phagolysosome maturation, escape into the cytosol, and manipulation of apoptosis or necrosis pathways, to transcend innate immune defenses and foster an environment conducive to its latent propagation.

### Neutrophils-mediated immunoreaction during *M. tuberculosis* infection

During *M. tuberculosis* infection, the host’s immune system mobilizes different myeloid lineage cells to combat the pathogen, including neutrophils, DCs, and various macrophage subsets, aside from AM. Neutrophils, known for their short lifespan, stand at the forefront of the host’s innate immune defenses as professional phagocytes (117). Although neutrophils are activated by *M. tuberculosis* and employ a range of antimicrobial effector mechanisms, these efforts fall short in controlling the infection. Instead, the interaction between neutrophils and *M. tuberculosis* frequently culminates in necrotic cell death of the neutrophils. Subsequently, these necrotic cells are phagocytosed by macrophages, a process which paradoxically fosters the proliferation of *M. tuberculosis* and precipitates the recruitment of additional neutrophils to the site of infection (118, 119).

## Adaptive immunity to *M. tuberculosis*

Following the initial implantation of bacteria into the pulmonary system, the priming of adaptive immune responses in the lung-draining lymph nodes typically commences within a two-week period. This pivotal process is subsequently followed by the infiltration of adaptive immune cells into lung tissue, which becomes actively engaged in combating the infection over the course of an additional one to two weeks. The initiation and orchestration of adaptive immune responses are fundamentally dependent on the early intervention of the innate immune system, underscoring its critical role in the host’s defense mechanism against *M. tuberculosis* infection.

### Cellular immunity in *M. tuberculosis* infection

In the investigation of cellular immunity against *M. tuberculosis* infection, Wolf et al. illuminated the critical role of DCs after exposing mice to GFP-expressing *M. tuberculosis*. They found that DCs, which are professional antigen-presenting cells (APCs), were frequently infected in the lungs and migrated to the draining lymph nodes (120). These cells are instrumental in eliciting adaptive immunity by presenting *M. tuberculosis* antigens via MHC-II and co-stimulatory molecules, thus initiating antigen-specific T cell responses.

The essential role of MHC-II-restricted CD4^+^ T cells in mounting a defense against tuberculosis becomes evident in individuals with HIV-related CD4^+^ T cell impairments, who exhibit a higher susceptibility to the disease (121). This significance is further underscored by studies utilizing CD4^+^ T cell depletion in non-human primates (NHP) and MHC-II deletion in mice (122, 123). While CD8^+^ T cells are vital for the immunological response to *M. tuberculosis*, their presence cannot compensate for the deficiency of CD4^+^ T cells (122). Mice deficient in the Th1-polarizing cytokine interleukin-12 (IL-12) or the transcription factor T-bet, responsible for specifying the Th1 lineage, succumb shortly after *M. tuberculosis* exposure, highlighting the dependence of host resistance to mycobacterial infection on Th1-oriented CD4^+^ T cells (124, 125). The arrest in bacterial growth is achieved when T cells, attracted to the lungs, interact with infected cells, recognize the antigens, and activate myeloid cells to halt the replication of ingested bacteria (125–127).

The study of individuals afflicted by diseases caused by less virulent mycobacteria reveals that genetic mutations impairing the IL12/IFN-γ pathway predispose individuals to mycobacterial infections (128). The pivotal effector mechanism of cell-mediated immunity is believed to be activated macrophages by type-1 Interferon γ (IFN-γ). This cytokine, primarily produced by natural killer (NK) and Th1 cells, is regulated by IL-12, secreted by dendritic cells and macrophages. IFN-γ, in concert with tumor necrosis factor α (TNF-α), triggers the microbicidal activities of macrophages essential for eliminating the intracellular pathogen.

CD8^+^ T cells also play a crucial protective role against mycobacterial infections. Mice lacking either TAP-1 (transporter associated with antigen processing 1) or β2 microglobulin, crucial components for MHC-I antigen presentation, display impaired CD8^+^ T cell responses and succumb more rapidly post-*M. tuberculosis* infection compared to wild-type controls (129, 130). Mott et al. have shown that high intracellular levels of *M. tuberculosis* induce macrophage death (131). This allows other macrophages to scavenge the resulting cellular debris and indirectly present the TB10.4 antigen to CD8^+^ T cells. CD8^+^ T cells’ ability to produce cytolytic effector molecules, such as perforin alongside IFN-γ and TNF-α, to lyse *M. tuberculosis*-infected macrophages, is vital for curbing bacterial proliferation (130). Moreover, CD8^+^ T cells’ capacity to release granulysin for direct bacilli destruction within macrophages underscores their importance in the immune response to *M. tuberculosis* (130, 132). Assistance from CD4^+^ T cells mitigate CD8^+^ T cell exhaustion and enhances their functional activities. The collaborative action of CD4^+^ and CD8^+^ T cells improve the survival outcomes of infected mice (133).

### Strategies of *M. tuberculosis* for cellular immune evasion

**Figure 4:**
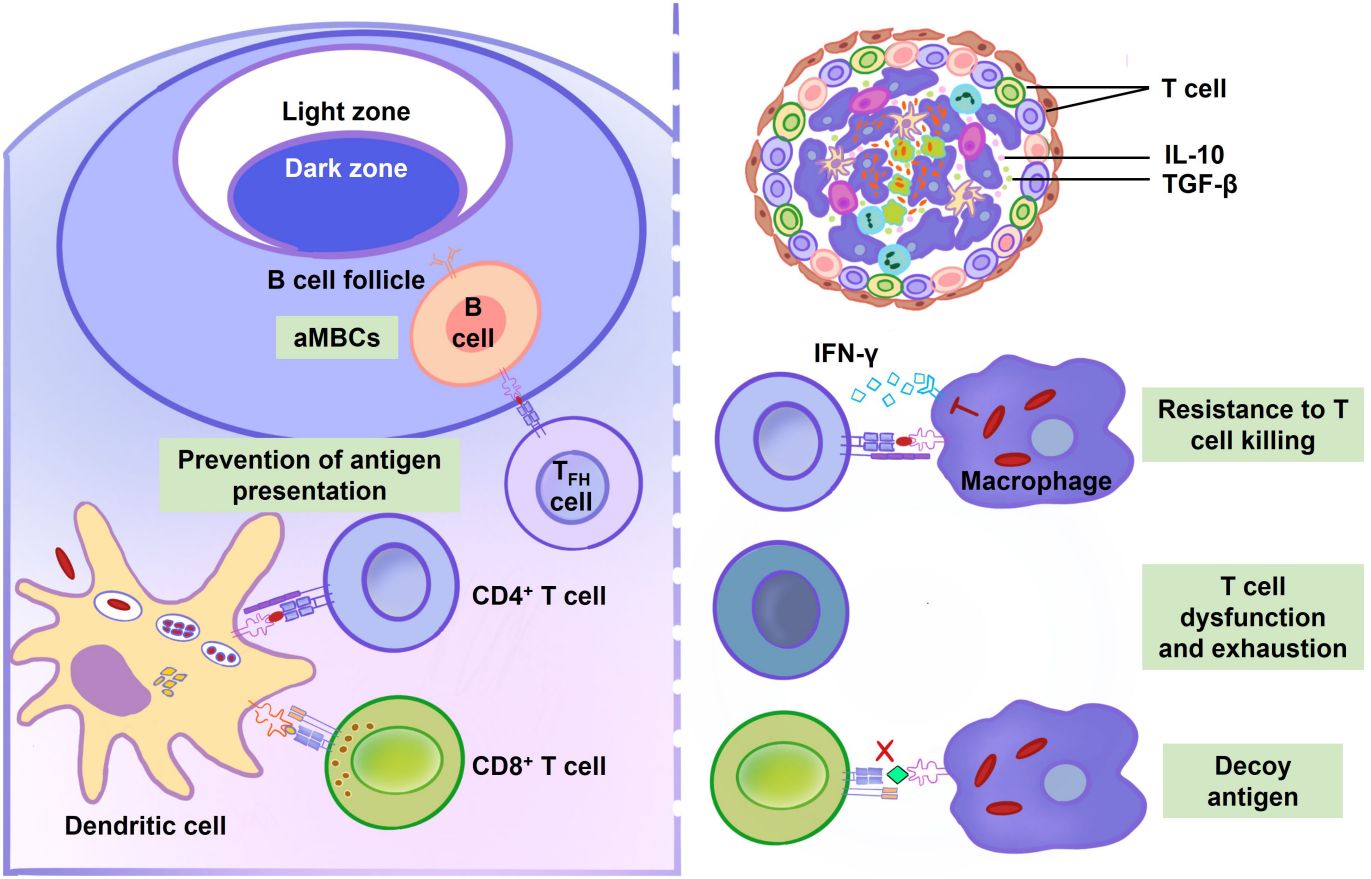
Adaptive immune response during *M. tuberculosis* infection. Dendritic cells (DCs) exhibit a delayed migration and antigen presentation to lymph nodes after *M. tuberculosis* infection. Compared to uninfected DCs, infected DCs are less efficient in stimulating T cells. GroEL2, PDIM and MPT64 suppress the maturation and activation of DCs. By preventing antigen presentation, EsxH, PE_PGRS47, 19-kDa lipoprotein, TDM, and NuoG postpone T cell activation. B cell function is also compromised during *M. tuberculosis* infection. Atypical memory B cells (aMBCs) express a variety of inhibitory receptors and resist antigen stimulation, making it difficult to stimulate them to replicate or release cytokines or antibodies. The proliferation of aMBCs obstructs the establishment of TB immunity. Additionally, it is difficult for T cells killing in granulomas because they are located far from infected center. IL-10 and transforming growth factor-β (TGF-β) play a role in the granuloma’s localized T cell suppression. Tryptophan can be produced by *M. tuberculosis* in order to resist T cells killing. Mycobacterial lipoglycans, mycolic acids, MPT70 and ESAT-6 cause T cells dysfunction and exhaustion. *M. tuberculosis* selectively elicits T cell responses to decoy antigens, which is poorly presented by infected cells.

*M. tuberculosis* employs a myriad of strategies to circumvent cellular immune defense mechanisms, resulting in substantial bacterial loads in the lungs even in the presence of robust adaptive immune responses. This evasion of cellular immunity is intricately illustrated in **Figure 4** and **Table 2**. The transport of *M. tuberculosis* from the pulmonary site to proximal draining lymph nodes is facilitated by DCs, which play a pivotal role in T cell activation during the infection (154). Instead of adhering to the canonical MHC-II antigen presentation pathway, infected DCs exploit a kinesin-2 dependent mechanism for antigen conveyance, thereby impairing the efficacy of CD4^+^ T cell stimulation (134). *M. tuberculosis* further undermines immune defenses by producing effector molecules that derail DC phagosome maturation and integrity, significantly hampering T cell priming.

**Table 2:** List of antigens by which *M. tuberculosis* overcomes adaptive immunity.

Overview	Effector	Mechanism	References
Delaying the development of adaptive immunity	Kinesin-2 of DCs	As bacterial antigens in *M. tuberculosis*-infected DCs are exported via kinesin 2-dependent vesicular transport rather than the MHC class II antigen presentation pathway, these DCs are less effective in stimulating T cells than uninfected DCs	(134)
	MPT64 (Rv1980c)	DCs exposed to MPT64 develop into myeloid-derived suppressor cells (MDSCs)	(135)
	PDIM	PDIM lowers the expression of CD86 and IL-12, suppresses the activation of DCs and macrophages, and prevents the priming and development of polyfunctional T cells	(136)
	GroEL2	The cleaved form of GroEL2, which is abundant in *M. tuberculosis*, is not able to promote the maturation of DCs or the presentation of antigen	(137)
	EsxH	EsxH prevented macrophages and DCs from stimulating CD4^+^ T lymphocytes	(138)
	PE_PGRS47	The PE_PGRS47 protein prevents mycobacteria-infected DCs from presenting antigen in an MHC class II-restricted pathway	(139)
	19-kDa lipoprotein	Through a mechanism depended on Toll-like receptor 2 (TLR2), 19-kDa lipoprotein inhibits MHC class II production and antigen processing, enabling *M. tuberculosis* to avoid detection by CD4^+^ T cells	(140)
	Trehalose 6,6’-dimycolate (TDM)	TDM on mycobacterial surface inhibits macrophage antigen presentation and reduces the expression of surface markers on macrophages	(141)
	NuoG	The inhibition of neutrophil apoptosis by NuoG leads to a delayed activation of CD4^+^ T cells	(142)
Resistance to T cells killing	Tryptophan	*M. tuberculosis* can produce tryptophan in response to stress to prevent amino acid starvation caused by T cells	(143)
The expression of immunodominant proteins by *M. tuberculosis* causes T cell exhaustion	ESAT-6	Functional exhaustion limits the ability of ESAT-6-specific T cells to provide protection	(144)
CD4^+^ T cells produce insufficient effects	Lipoarabinomannan and other mycobacterial lipoglycans	T cell responses are inhibited by *M. tuberculosis* lipoglycans that are carried by bacterial vesicles and released from infected macrophages	(145)
	Mycolic acids	Mycobacterial mycolic acids bind to the inhibitory receptor Clec12A to limit T cell responses	(146)
	MPT70	IFN-γ causes MPT70 level to increase in infected macrophages, and high MPT70 expression results in highly differentiated CD4^+^ T cells with reduced protective potential	(147)
Expression of decoy antigens by *M. tuberculosis* did not confer protection	TB10.4	Decoy antigen TB10.4 impedes the expansion of CD8^+^ T lymphocytes that identify mycobacterial epitopes provided by *M. tuberculosis*-infected macrophages	(148)
	Ag85B	Ag85B-specific T cells first proliferate before beginning to decline four weeks after the infection. The pathogen’s low expression of Ag85B limits the protective ability of Ag85B-specific T cells	(144)
Inhibition of T cell function (in the TB granuloma)	IL-10	Protective immunity is inhibited by IL-10, which is generated by macrophages, neutrophils, CD4^+^ T cells and Tregs	(149)
	TGF-β	TGF-β limits T cell proliferation, survival, and function inside the tuberculosis granuloma	(150)
	Indoleamine 2,3-dioxygenase (IDO) enzyme	By blocking IDO activity, granuloma organization is changed, causing a greater number of T cells to migrate to the lesion core and display high proliferation	(151)
Inhibition of B cell function	B cell	B cell function is damaged during active TB and latent TB infection, and this B cell dysfunction weakens cellular host immunity	(152)
	Atypical memory B cells (aMBCs)	The growth of aMBCs is stimulated by TB, and it is possible that aMBCs impede the development of immunity against TB	(153)

Saini et al., through genome-wide analysis, spotlight the mycobacterial PE_PGRS47 protein as an autophagy inhibitor that constrains the delivery of MHC-II-restricted antigen by *M. tuberculosis*-infected dendritic cells (139). Additional hurdles, such as the pathogen’s slow replication rate, its inhibition of host cell apoptosis, and the sluggish activation and migration of DCs, collectively contribute to the delayed initiation of adaptive immunity. These elements synergistically facilitate the establishment and persistence of *M. tuberculosis* infection within the lungs.

Despite prompt lung infiltration by antigen-specific T cells, their attempts to clear the infection are thwarted by *M. tuberculosis* through multiple mechanisms. Effective suppression of intracellular *M. tuberculosis* necessitates the direct recognition of infected cells by CD4^+^ T cells (155). Yet, the physical separation between T cells and their infected targets often presents a significant obstacle. Furthermore, membrane vesicles containing *M. tuberculosis* cell envelope lipoglycans, released by infected macrophages, can impede CD4^+^ T cell activation, as evidenced by diminished interleukin-2 (IL-2) secretion and impaired T cell proliferation (145).

Another challenge arises when CD4^+^ T cells encounter consistently expressed antigens like ESAT-6, leading to their proliferation within the lung parenchyma and subsequent functional exhaustion or progression towards terminal differentiation, diminishing their defensive capabilities (144). *M. tuberculosis* strategically redirects T cell focus towards immunodominant, yet nonprotective antigens that are ineffectively presented by infected cells (148).

In summation, the adaptive immune response against *M. tuberculosis* is significantly delayed and inadequate, characterized by an orchestrated evasion strategy that leverages both the manipulation of antigen presentation and the impairment of T cell function, thereby enabling the pathogen’s sustained survival within the host.

### Humoral immunity in *M. tuberculosis* infection

An increasing body of research suggests that antibodies and B cells may confer protection against *M. tuberculosis* infection (156–162). The role of humoral immunity in the context of *M. tuberculosis* infection has been substantiated by a plethora of serological studies, which reveal that despite being an intracellular bacterium, *M. tuberculosis* elicits a comprehensive humoral response against a diverse array of mycobacterial antigens in humans (163). Animal models deficient in humoral immunity and B cell function exhibit increased susceptibility to tuberculosis infection, underscoring the importance of these immune components (161, 164–167). Additionally, the passive transfer of monoclonal antibodies targeting *M. tuberculosis* has been shown to ameliorate infections in mice, further supporting their protective role (168–180). In humans, elevated titers of certain IgG are typically observed in tuberculosis patients. However, the absence of galactose in the carbohydrates linked to the Fc region of IgG impairs the protective function of these antibodies, as demonstrated by Olivares et al., where mice treated with deglycosylated IgG did not exhibit protection (181). This finding indicates that IgG glycosylation is necessary for immunoglobulin to confer a protective effect against TB in mice.

Antibody-mediated opsonization is believed to confer protective benefits at the onset of infection. This is predominantly achieved through the enhancement of mycobacterial antigen uptake by phagocytic cells (160). The process is facilitated via Fc receptor (FcR)-mediated phagocytosis, which subsequently leads to an increase in intracellular pathogen elimination (182).

In addition to providing direct assistance with phagocytosis, antibodies can modulate the complement system, thereby catalyzing inflammation and further enhancing phagocytic activity (183). During complement receptor-mediated phagocytosis of *M. tuberculosis*, altered Ca^2+^ signaling has been observed. The suppression of this Ca^2+^ signaling pathway by *M. tuberculosis* indicates that such alterations in macrophage activation significantly contribute to the inhibition of phagosome-lysosome fusion, consequently promoting the intracellular survival of the mycobacteria (184).

Furthermore, antibodies targeting the cell-surface lipopolysaccharide of *M. tuberculosis* may expedite pathogen clearance, thereby attenuating the possibility of detrimental interference with immune responses. The mycobacterial surface material lipoarabinomannan (LAM) has been associated with several detrimental effects on immune system function. In mice, LAM-binding antibodies significantly reduced the amount of LAM deposited in the spleen, suggesting that these antibodies may play a role in altering the course of mycobacterial infection (171, 185).

Antibodies carry with them the dual capability to either dampen or amplify inflammatory responses due to their constituents of pro- and anti-inflammatory molecules (186). This multifaceted functionality permits antibodies to regulate the intensity of the immune response, potentially mitigating the tissue-damaging consequences of uncontrolled granuloma—a process intricately described in subsequent sections. Pro-inflammatory antibodies can assist hosts with insufficient inflammatory responses, while anti-inflammatory antibodies may benefit hosts experiencing excessive inflammation that leads to tissue damage (156).

In summary, the humoral immune response against *M. tuberculosis* is marked by a multilayered mechanism involving phagocytic enhancement, complement activation, pathogen clearance, and modulation of the inflammatory milieu. This intricate interplay between various antibody-mediated activities plays an essential role in shaping the course and outcome of *M. tuberculosis* infection.

### Strategies of *M. tuberculosis* for humoral immune evasion

Recent investigations have unveiled novel strategies by which *M. tuberculosis* subverts humoral immunity, particularly impacting the functional capacity of certain B lymphocytes—a phenomenon comprehensively illustrated in **Figure 4** and **Table 2**. These B cells manifest a compromised state evidenced by their diminished cytokine secretion, immunoglobulin production, and proliferative ability (152). This dysfunctional B cell phenotype, frequently termed ‘atypical’, has been observed in the milieu of various infectious diseases, notably chronic or recurrent infections such as malaria, and infections caused by hepatitis B and C viruses, as well as HIV (153, 187, 188).

The role of atypical B cells within the immune landscape remains an enigma, yet they are hypothesized to exert detrimental effects on pathogen-specific immune responses. The concerns pivot around the observed elevated concentrations of these cells during infection, which may signify a potential hindrance to the efficacy of the humoral response (153). As such, mycobacterial ability to induce and sustain the proliferation of atypical B cells represents another facet of its immune evasion repertoire, possibly allowing the pathogen to persist by attenuating the protective functions typically mediated by conventional B lymphocytes.

## Formation of the granuloma

**Figure 5:**
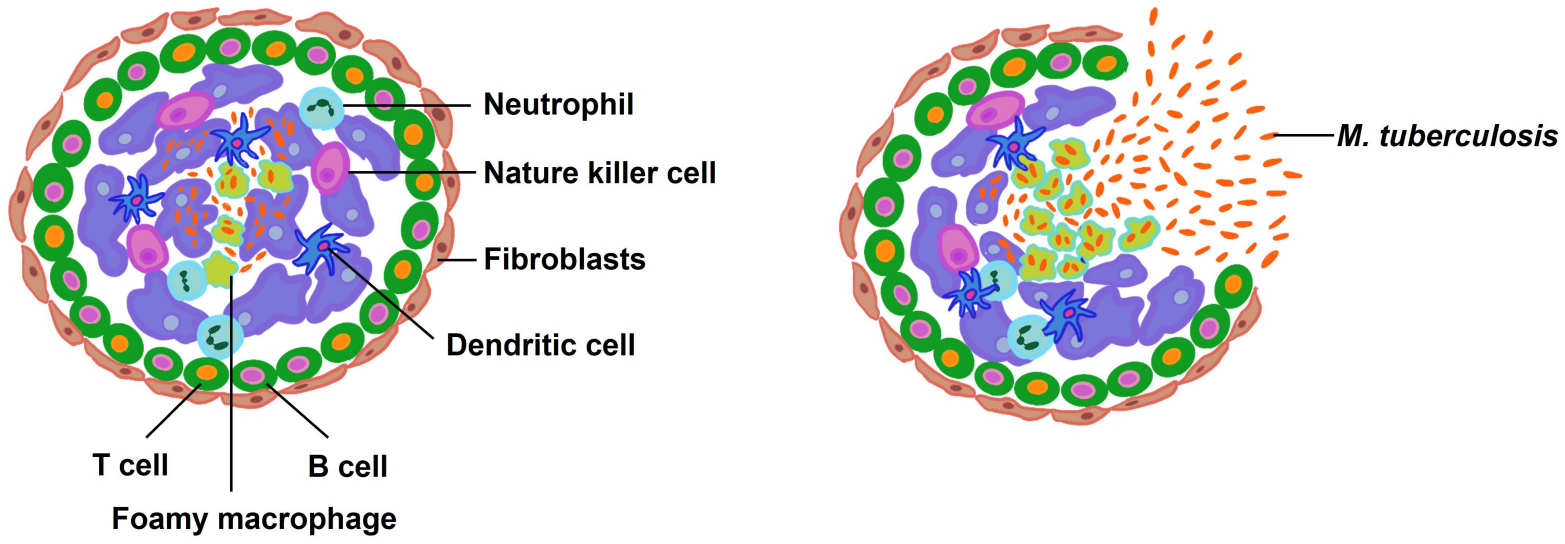
The composition and rupture of granuloma. Macrophages consume the bacilli when they enter the lung. In order to sterilize the infected macrophages, more immune cells are enlisted, which causes the granuloma formation. Latent infection persists in healthy individuals but reactivation is possible. When *M. tuberculosis* is reactivated for some reason, the bacteria multiply and the bacterial load rises to an unmanageable level. At this point, the granuloma bursts, releasing the bacteria into the airways.

The formation of the granuloma constitutes a hallmark histological feature of tuberculosis infection. Granulomas are structured aggregates predominantly comprised of both infected and uninfected macrophages at various stages of differentiation (as depicted in **Figure 5**) (189–191). Within these complex formations, macrophages undergo epithelioid transformation, become lipid-laden foamy cells, or coalesce into multinucleated giant cells. This central macrophage hub is encircled by a heterogeneous population of immune cells including neutrophils, DCs, T cells, B cells, and fibroblasts. Gradual development of hypoxia within the granuloma culminates in necrotic death of cells, leading to the formation of a necrotic cell-free zone known as the caseum (192).

Granulomas serve a dual role; they are essential host defense structures that contain the bacterial spread and also provide a niche wherein *M. tuberculosis* can reside and potentially persist in a dormant state until conditions favor reactivation and proliferation. While the immune response helps to eradicate bacteria from some granulomas, in others, the bacteria can survive and proliferate. Early granulomas with high bacterial loads are influenced by mast cells, type 2 immunity, and tissue remodeling. In contrast, low-burden, late-forming granulomas are associated with a response involving cytotoxic T cells and type 1-type 17 immunity (193).

Immunologically, granulomas are characterized by an inhibitory milieu, with the immune-regulatory cytokine IL-10 implicated in compromising T cell activity within this environment (149, 194, 195). Moreover, T cell suppression within the granuloma is further enhanced by transforming growth factor-β (TGF-β). Disruption of TGF-β signaling in T cells has been shown to augment IFN-γ production and enhances T cell accumulation within granulomas (150). Notably, CD4^+^ T cells are predominantly localized at the periphery of granulomas, maintaining a distance from the centrally located infected macrophages, potentially limiting their effectiveness in controlling the infection (150, 151).

Mycobacterial lipids play a pivotal role in the pathogenesis of granuloma formation. They not only impair the antimicrobial capabilities of macrophages but also promote the recruitment of additional macrophages, ultimately facilitating the dissemination of *M. tuberculosis* (196). Granulomas harboring *M. tuberculosis* can remain stable for extended periods, often spanning decades (197). However, factors such as the organism’s capacity for immunosuppression can jeopardize the granuloma’s integrity, potentially leading to its rupture and consequent dissemination of a substantial number of viable bacilli.

## Pulmonary and extrapulmonary tuberculosis

The clinical manifestations of TB are contingent upon the anatomical site of the *M. tuberculosis* proliferation within a host. As an obligate aerobe, *M. tuberculosis* exhibits a predilection for the pulmonary system, whereby pulmonary TB can manifest as persistent cough, often accompanied by hemoptysis or sputum production, chest pain, and a protracted cough persisting beyond three weeks. General systemic symptoms attributable to TB may include malaise, anorexia, significant weight loss, febrile episodes, chills, and nocturnal hyperhidrosis. Other organs afflicted by *M. tuberculosis* may present with localized symptoms reflective of the pathogen’s dissemination.

Pulmonary immune responses elicited by mycobacterial infection are characterized by the activation of AMs, DCs, and inflammatory mediators, which in turn recruit and activate monocytes, neutrophils, T cells, and B cells. These inflammatory events culminate in granuloma formation. However, the persistent inflammatory response associated with *M. tuberculosis* infection provokes tissue necrosis, chronic lung inflammation, airway remodeling, and fibrotic alterations (198–204). The ensuing chronic obstructive pulmonary pathology ultimately culminates in irreversible airflow obstruction, potentially escalating to chronic respiratory failure and mortality (205, 206).

**Figure 6:**
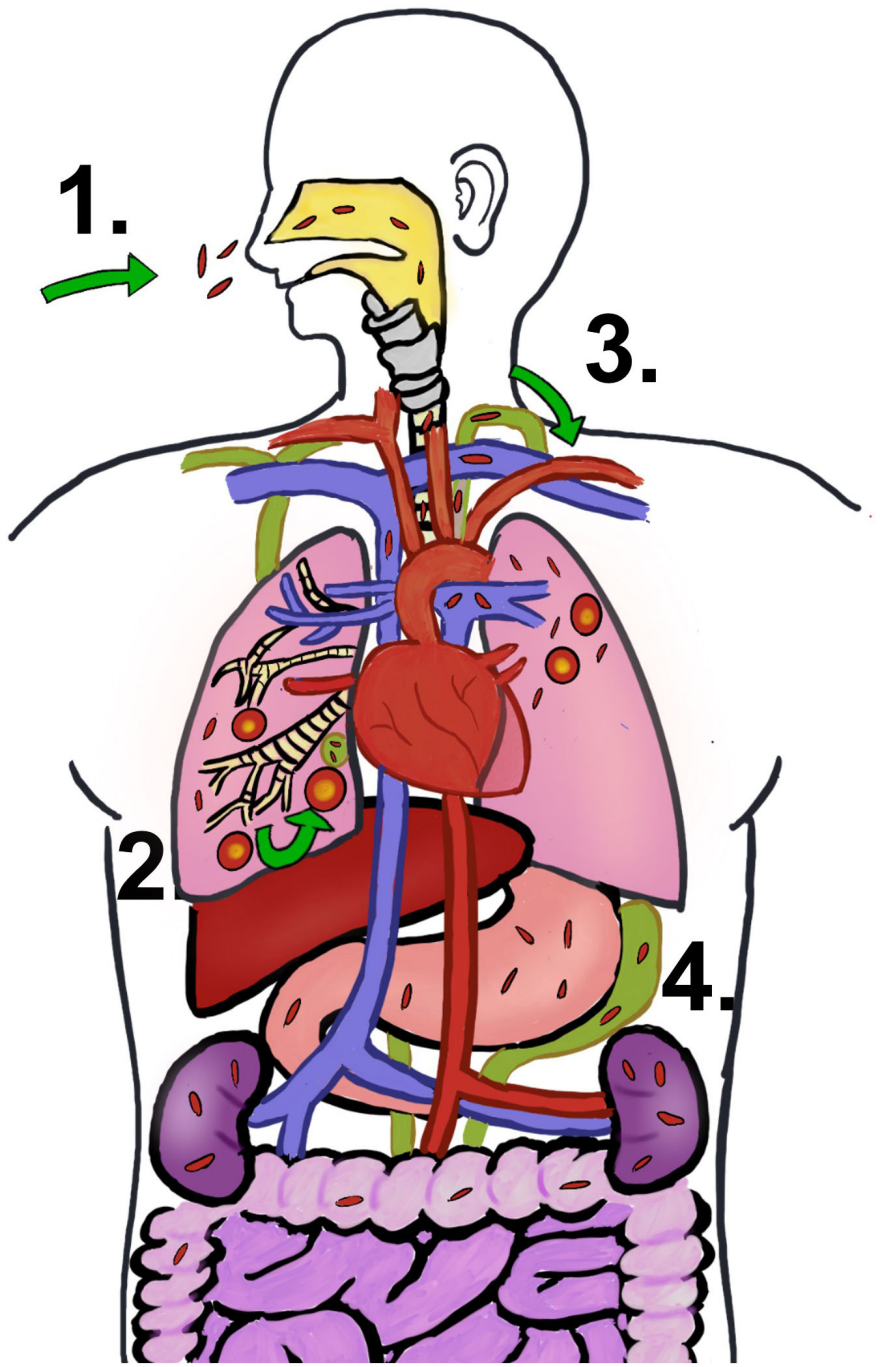
*M. tuberculosis* infection progression. (1) People get TB by breathing in the bacteria’s air. (2) Initial infection is developed in lung and go on to become granuloma. The early infection site impacts the surrounding lymph nodes as well. (3) Once the bacteria leave the lung and enter the lymphatic system, they most likely find their way into the circulatory system via the thoracic duct’s entrance into the subclavian vein. (4) Extrapulmonary TB is caused by the hematogenous spread of *M. tuberculosis*.

Beyond the pulmonary confines, *M. tuberculosis* can travel through the blood or lymphatic system from the primary lung lesions to disparate organ systems—this process characterizes extrapulmonary TB, accounting for approximately 15% of TB cases (207). Following the initial aerosolized infection and lung invasion, *M. tuberculosis* colonizes adjacent lymph nodes before gaining access to the bloodstream via the lymphatic network (illustrated in **Figure 6**) (208). Both immunocompetent and immunocompromised individuals are susceptible to extrapulmonary TB, although individuals living with HIV bear a heightened risk for extrapulmonary manifestations, particularly within the lymphatic system, with widespread dissemination, and affecting the central nervous system (CNS) (209). This complex pathology underscores the necessity for robust diagnostic and treatment strategies to mitigate the extensive spectrum of TB disease.

## Designing tuberculosis vaccines based on pathogenesis

The global tuberculosis vaccine pipeline currently includes 17 candidates undergoing clinical trials, which we have organized and evaluated in **Table 3**. Although many of these candidates have successfully entered Phase I clinical trials, only a limited number have progressed to large-scale studies (227). A significant factor contributing to this limited advancement is the diminished efficacy of the vaccines.

**Table 3:** The TB vaccine pipeline 2024.

Type	Number	Name	Stage	Sponsor	Antigen	Vector	Adjuvant	Evaluation	References
Inactivated vaccines	1	Immuvac (MIP)	III	Indian Council of Medical Research, Cadila Pharmaceuticals	*M. indicus pranii*	Not Available	Not Available	Not Available	(210)
	2	RUTI	IIb	Archivel Farma, S.L	Liposome suspension of mycobaterial cell wall nanofragments	Not Available	Not Available	The RUTI vaccine shows acceptable safety and promising immunogenicity but requires further research to address local adverse reactions and optimize immune response.	(211)
	3	DAR-901	IIb	Dartmouth, St. Louis University	Inactivated *M. obuense*	Not Available	Not Available	DAR-901 primarily induced Th1 responses. Although DAR-901 responses were more moderate, BCG generated greater CD4^+^ T cell responses than placebo. A three-dose course of 1 mg DAR-901 did not stop initial or ongoing Interferon Gamma Release Assay (IGRA) conversion.	(212–214)
Live attenuated vaccines	1	MTBVAC	III	Biofabri, Bharat Biotech, University of Zaragoza, IAVI, TBVI, HIV Vaccine Trials Network	Mtb103 without phoP (Rv0757) and fadD26 (Rv2930)	Not Available	Not Available	MTBVAC demonstrates promising safety and immunogenicity profiles in both adults and infants, suggesting potential as a future tuberculosis vaccine candidate.	(215)
	2	BCG (Revaccination)	IIb	Bill & Melinda Gates Medical Research Institute	BCG	Not Available	Not Available	The effectiveness of the H4:IC31 vaccination was 30.5% (P=0.16), but the BCG vaccine had an efficiency of 45.4% (P=0.03) in reducing the rate of sustained QuantiFERON-TB Gold In-tube assay (QFT) conversion.	(216)
	3	BCG (Travel vaccine)	III	Henry M. Jackson Foundation for the Advancement of Military Medicine	BCG	Not Available	Not Available	The aim of this study is to determine if adult travelers to regions with high TB rates can reduce their risk of contracting TB by eliciting an immunological response after receiving a single dose of the BCG pre-travel immunization.	(210)
	4	VPM1002	III	Serum Institute of India Private Limited, Vakzine Projekt Management GmbH	VPM1002 is a recombinant BCG vaccination strain that expresses listeriolysin and lacks urease C	Not Available	Not Available	VPM1002 demonstrates superior safety compared to BCG, with significantly lower rates of severe adverse reactions such as lymphadenopathy and injection site complications. While slightly less immunogenic than BCG, VPM1002 shows promising results and is advancing to phase 3 trials for further assessment of efficacy and safety in infants in sub-Saharan Africa.	(217)
Subunit vaccines	1	M72/AS01E	III	Bill & Melinda Gates Medical Research Institute, GSK Vaccines	Mtb32A-Mtb39A	Not Available	MPL+QS-21+cholesterol (AS01E)	M72/AS01E vaccine showed about 50% protection against TB in adults infected with *M. tuberculosis* over 3 years, suggesting it could be valuable for global TB control. The immune responses it triggered lasted up to 36 months, supporting its potential in future studies involving diverse populations.	(218)
	2	GamTBvac	III	Gamaleya Res. Centre, MoH Russia	DBD-Ag85a, DBD-ESAT6-CFP10	Not Available	Dextran 500 kDa and DEAE-Dextran 500 kDa coated with CpG oligonucleotides are included in the adjuvant	The GamTBvac vaccine demonstrated tolerability and exhibited an acceptable safety profile. The findings of antigen-specific IFN-γ release, Th1 cytokine-expressing CD4^+^ T-cells, and IgG responses from the vaccine support further clinical testing of GamTBvac.	(219)
	3	ID93+GLA-SE (QTP101)	IIa	NIAID, NIH	Rv1813, Rv2608, Rv3619, Rv3620	Not Available	Glucopyranosyl lipid adjuvant (GLA)-stable emulsion (SE)	In adult healthcare workers who had not previously infected *M. tuberculosis* but had received a BCG vaccination, the ID93 + GLA-SE vaccine elicited antigen-specific cellular and humoral immune responses with a tolerable safety profile.	(220)
	4	AEC/BC02	IIa	Anhui Zhifei Longcom Biopharmaceutical Co., Ltd.	Ag85b, ESAT6-CFP10	Not Available	Cytosine guanine dinucleotide (BCG-cpg-DNA) and aluminum hydroxide of BCG (BC02)	In mice, the AEC/BC02 vaccination produced robust cellular immunological responses, as evidenced by a high frequency of antigen-specific T cells that secrete IFN-γ.	(221)
	5	H107e/CAF10b	I	Statens Serum Institut	PPE68/Rv3873, ESAT-6/Rv3875, EspI/Rv3876, EspC/Rv3615c, EspA/Rv3616c, MPT70/Rv2875, MPT83/Rv2873	Not Available	CAF^®^10b	Unlike BCG, H107e possesses eight distinct protective antigens. Mice were able to develop long-lasting immunity and Th17 responses specific to BCG after receiving a single dosage of H107e/CAF^®^01 combined with BCG.	(222)
Viral vectored vaccines	1	AdHu5Ag85A	I	McMaster University, CanSino	Ag85A	Adenovirus	Not Available	Aerosol delivery of AdHu5Ag85A vaccine is safe, induces robust respiratory mucosal immunity, and elicits systemic T cell responses, highlighting its potential for developing effective aerosol vaccine strategies against respiratory pathogens like TB and COVID-19.	(223)
	2	TB/FLU-05E	I	Smorodintsev Research Institute of Influenza, Ministry of Health of the Russian Federation	Truncated NS1 protein NS1(1–124), TB10.4, HspX	Recombinant attenuated influenza vector (Flu/THSP)	Not Available	A BCG-prime and Flu/THSP vector boost vaccination system was demonstrated to shield mice against *M. tuberculosis*-induced severe lung damage because it boosted the T-cellular immune response, which is mediated by antigen specific CD4^+^ and CD8^+^ T-lymphocytes.	(224, 225)
	3	ChAdOx1.85A+MVA85A	IIa	University of Oxford	Ag85A	Simian adenoviral vector (ChAdOx1.85A), modified vaccinia virus Ankara (MVA85A)	Not Available	ChAdOx1.85A+MVA85A were safe for adults and evoked polyfunctional CD4^+^ T cells (IFN-γ, TNF-α, and IL-2) as well as IFN-γ^+^, TNF-α^+^ CD8^+^ T cells and Ag85A IgG responses.	(226)
mRNA vaccines	1	BNT164a1	I	BioNTech, Gates foundation	Not Available	Not Available	Not Available	Not Available	(210)
	2	BNT164b1	I	BioNTech, Gates foundation	Not Available	Not Available	Not Available	Not Available	(210)

To effectively counter TB, vaccine development must be intricately informed by a nuanced understanding of the pathogenesis of *M. tuberculosis*. An optimized vaccine would preemptively hinder *M. tuberculosis* from entering host cells, amplify both innate and adaptive immune responses, and surmount the bacterium’s immune evasion tactics. Nearly all of the antigens known to be involved in the pathogenic process of *M. tuberculosis* are summarized in **Tables 1**, **2**. The development of new tuberculosis vaccines can consider combining these appropriate antigens.

The interplay of surface adhesion molecules is crucial for the invasion of host cells by mycobacteria, facilitating their association with and entry through host cellular receptors (228, 229). Various adhesion molecules, such as HBHA and fibronectin-binding proteins (FnBPs), significantly contribute to bacterial colonization by promoting the internalization of *M. tuberculosis* into host cells (230). The application of anti-HBHA antibodies to wild-type mycobacteria significantly inhibits their ability to disseminate following intranasal infection (231). These interactions with host cells not only aid in attachment and invasion but also trigger a series of signaling cascades, including the activation of the mitogen-activated protein kinases (MAPKs) pathway and the IFN-γ response, which can elicit pro-inflammatory and/or anti-inflammatory events (229). Moreover, adhesion molecules interfere with host signaling pathways and modify intracellular mechanisms, thereby modulating the immune response. Consequently, targeting these molecules is pivotal for the development of innovative tuberculosis vaccines and therapeutics.

Augmenting the bactericidal activity of primary mycobacterial targets—innate immune cells—stands as a critical strategy for curtailing *M. tuberculosis* proliferation. Trained immunity refers to a heightened state of innate immunological readiness that confers prolonged protection (232). Evidence suggests this heightened readiness can preserve its protective efficacy for months, if not years, following exposure to live vaccines (233, 234). Epigenetic and metabolic reprogramming of monocytes and macrophages constitute the mechanistic underpinnings of trained immunity, bolstering mycobacterial eradication and fostering expeditious clearance (235). β-glucan, renowned for immune potentiation in cancer therapy, exemplifies an agent that has shown promise in clinical trials for enhancing resistance to *M. tuberculosis* infection by stimulating myeloid progenitor proliferation (236–240). Similarly, the BCG vaccine can induce preventive innate immunity through the reprogramming of hematopoietic stem cells towards myelopoiesis in the bone marrow (241). The exploration of vaccine candidates that amplify innate immunity and elicit trained immunity could revolutionize TB vaccine efficacy.

DCs serve as a linchpin in the host’s defensive apparatus as primary antigen-presenting cells. Bolstering DCs through vaccines and immunotherapies could potentiate TB mitigation efforts. Enhancement of glutathione levels within DCs kickstarts the NF-κB signaling pathway, augmenting DC functionality in their dual roles of mycobacterial containment and antigen presentation (242). Exogeneous CD40 stimulation in infected DCs has been observed to amplify DC efficacy and boost CD4^+^ T cell responses, which aids in managing pulmonary bacterial loads (243). The introduction of antigen-loaded DCs has demonstrated the potential to enhance the efficacy of BCG vaccines, accentuating the imperative role of DCs in vaccine success (244).

Immunological research has demonstrated the critical importance of CD4^+^ Th1 cells and cytokines such as IFN-γ, TNF-α, and IL-2 in orchestrating host immunity (245). By elucidating the pathogenesis of *M. tuberculosis*, researchers can more accurately identify antigens that instigate robust T cell responses, necessitating continued exploration for novel mycobacterial antigens through bioinformatics and genomics. In the field of immunoinformatics, computational techniques are employed to address immunological complexities, enabling the discovery of immunogenic T-cell peptides from *M. tuberculosis*, specifically MPT64, PPE68, CFP21, and Ag85B (246–250). These peptides have shown promising immunogenicity in laboratory studies and represent candidates for potential inclusion in future tuberculosis vaccines. Furthermore, unraveling the mechanisms behind the generation and maintenance of memory T cells is crucial for the development of effective T cell-targeted vaccines (251).

Recent studies have posited a reinforcing role for antibodies in the defensive cohort against *M. tuberculosis*, with antibodies against capsular polysaccharides conferring protection in various microbial infections (252). The capsule constituent arabinomannan (AM) plays a critical role in TB pathophysiology (253), and vaccinations using AM conjugates have conferred heightened resistance in murine models (254, 255). HBHA also plays a critical role in the dissemination of mycobacteria. Studies have shown that coating BCG with anti-HBHA antibodies significantly reduces spleen colonization, suggesting that targeting HBHA can be an effective strategy to limit the spread of mycobacterial infections (231). In mycobacterium-infected mice, intranasal administration of an IgA monoclonal antibody targeting the α-crystallin antigen of *M. tuberculosis* resulted in a significant tenfold reduction in bacterial counts within the lungs (174). Furthermore, BCG and several other antigen-based vaccines elicit humoral responses that enhance TB prognoses (156).

Confronting mycobacterial adept immune evasion strategies is another key to vaccine advancement. *M. tuberculosis* employs mechanisms to dampen immune responses, devastate immune cells, and diminish metabolic visibility for persistent cellular habitation (**Tables 1**, **2**). Targeted disruption of these mycobacterial evasion pathways will substantially amplify vaccine potency. For instance, the ESX-1 type VII secretion system is implicated in facilitating mycobacterial cellular escape and dissemination, and its incorporation into the BCG genome—yielding BCG::ESX-1—has shown enhanced protective capacity against TB (256). The major secretory antigen, the antigen 85 (Ag85) complex, comprising Ag85A, Ag85B, and Ag85C, plays a crucial role in the pathogenicity of *M. tuberculosis*. This complex impedes the formation of phagolysosomes, thereby enabling the bacteria to overcome the host immune response and persist within host cells. Given its significant impact on the infection process, Ag85 molecules are being utilized in diagnostic procedures and the development of novel vaccines (257). ESAT-6 reduces T cell activation without altering upstream T cell receptor (TCR) signaling processes, thereby significantly inhibiting T cell production of IFN-γ in response to *M. tuberculosis* or TCR activation (258). ESAT-6 is currently utilized in tuberculosis-related vaccines, including AEC/BC02 and GamTBvac. The antigen TB10.4 is expressed by both *M. tuberculosis* and BCG. Upon activation, CD8^+^ T cells specific to the TB10.4 3–11epitope are recruited to the site of infection following *M. tuberculosis* exposure. These cells produce TNF-α and IFN-γ, and exhibit upregulated expression of Fas ligand (FasL) and lysosomal-associated membrane proteins 1 and 2 (LAMP-1/2, also known as CD107A/B) (259). The TB-related vaccine TB/FLU-05E, currently in Phase I clinical trials, employs TB10.4 as one of its antigens.

Collectively, these insights point towards a multifaceted approach to TB vaccine development, encompassing the targeting of adhesion molecules, fortification of innate immune responses, enhancement of DC functionality, discovery of novel antigens, and interruption of *M. tuberculosis* immune evasion mechanisms. Such a strategy presages advancements in durable and effective vaccines against TB.

## Conclusions

This review sheds light on the mechanisms by which *M. tuberculosis* enters host cells, disseminates infection, and evades the immune system. The vaccines for the future will need to overcome myeloid cell malfunction and the inability of the innate and adaptive immune systems caused by *M. tuberculosis* in order to eradicate the bacilli. The knowledge of pathogenesis should enable novel methods to produce effective vaccines, despite the numerous gaps that still need to be filled.

## Author contributions

HY: Writing – original draft, Writing – review & editing. XL: Writing – review & editing. SC: Writing – review & editing. GS: Writing – review & editing. LD: Writing – review & editing, Supervision.
